# Retraction Note: Precise determination of graphene functionalization by in situ Raman spectroscopy

**DOI:** 10.1038/s41467-022-34281-x

**Published:** 2022-11-04

**Authors:** Philipp Vecera, Julio C. Chacón-Torres, Thomas Pichler, Stephanie Reich, Himadri R. Soni, Andreas Görling, Konstantin Edelthalhammer, Herwig Peterlik, Frank Hauke, Andreas Hirsch

**Affiliations:** 1 Chair of Organic Chemistry II and Joint Institute of Advanced Materials and Processes (ZMP), Friedrich-Alexander University of Erlangen-Nuremberg, Henkestrasse 42, 91054 Erlangen, Germany; 2 Institut für Experimental Physik, Freie Universität Berlin, Arnimallee 14, 14195 Berlin, Germany; 3 Yachay Tech University, School of Physical Sciences and Nanotechnology, Urcuquí, 100119 Ecuador; 4 Faculty of Physics, University of Vienna, Strudlhofgasse 4, A-1090 Vienna, Austria; 5 Chair of Theoretical Chemistry, Friedrich-Alexander University Erlangen-Nürnberg (FAU), Egerlandstraße 3, 91058 Erlangen, Germany

Retraction to: *Nature Communications* 10.1038/ncomms15192, published online 08 May 2017

The authors have retracted this article because of data processing errors that impacted data presentation and interpretation of the Raman spectra plotted in Fig. 1c, Fig. 2b, and Fig. S2, which invalidate the conclusions of the article.

The authors state that the data processing was performed with a routine implemented in the OriginPro® software. The evolution of the Raman spectra upon exposure to an electrophilic trapping reagent (H_2_0 vapour, H_2_, O_2_) was displayed as cumulative plots, whereby individual spectra are added up consecutively and displayed in a ‘waterfall’ presentation. Upon re-examination of the data, the authors found that the C_z_ mode, characteristic of graphitic intercalation, vanishes with the continuous exposure of the electrophilic trapping reagent, whereas in the original Fig. 1c, Fig. S2 it is widely retained, and in some series it becomes more intense. The apparent persistence of the C_z_ mode is a direct consequence of the erroneous data processing. Any conclusions on the structure of the intercalated graphite during gas exposure that were drawn from the analysis of the C_z_ mode are invalid. Especially, pathway i) in Fig.2a of the paper is not supported by the measured data. Furthermore, the authors found that the spectra displayed in Fig. 1d, Fig. 2b (grey line) and Fig. 4b are identical, although they are described as different data sets, invalidating the analysis of the D bands.

All authors agree to this retraction.

